# Apalutamide: A Cause of Neutropenic Sepsis

**DOI:** 10.7759/cureus.75915

**Published:** 2024-12-17

**Authors:** Urwah Ali, Omar Khan, Venga Vengadasalam

**Affiliations:** 1 Oncology, The Great Western Hospitals National Health Services (NHS) Foundation Trust, Swindon, GBR; 2 Oncology, Oxford University Hospitals National Health Services (NHS) Foundation Trust, Oxford, GBR

**Keywords:** apalutamide, febrile neutropenia, platelets count, prostate cancer, psa level

## Abstract

Prostate cancer is one of the most frequently diagnosed cancers and poses a significant health burden. New androgen-targeted therapies are now standard treatments for various stages of prostate cancer, including hormone-sensitive, metastatic, and non-metastatic castration-resistant types. These therapies are generally well tolerated and often have fewer side effects compared to traditional chemotherapy.

Neutropenia and myelosuppression are considered very rare events with such medication and are more commonly associated with cytotoxic chemotherapy, such as docetaxel. Neutropenic sepsis is a serious condition that requires prompt recognition and aggressive management. Healthcare providers should be vigilant in monitoring blood counts and educating patients on the signs of infection to mitigate the risks associated with this potentially life-threatening complication which include acute kidney injury (AKI), hepatic encephalopathy, heart failure, central nervous system dysfunction, acute respiratory distress syndrome (ARDS) or acute respiratory failure, coagulopathy, and death.

Here, we describe the case of a 65-year-old man with metastatic hormone-sensitive prostate cancer who experienced neutropenic sepsis and myelosuppression as a result of treatment with the newer agent apalutamide. To our knowledge, this is only the second documented case of such side effects and the first to involve myelosuppression.

## Introduction

Prostate cancer ranks as the second most commonly diagnosed cancer among men globally, following lung cancer. In 2020 alone, approximately 1.4 million new cases were identified, underscoring the significant public health impact of this disease. It caused approximately 375,000 deaths, accounting for 6.8% of cancer-related deaths in males that year [1].

Androgen receptor targeting agents (ARTAs) are designed to block androgen receptors, which are critical for the growth of prostate cancer cells. Testosterone plays a significant role in the development and progression of prostate cancer as it stimulates the growth of cancer cells through androgen receptor activation. Apalutamide is an antiandrogen and acts as an antagonist to androgen receptors, preventing the effects of androgens like testosterone and dihydrotestosterone in the prostate gland and elsewhere in the body [2].

The Selective Prostate Androgen Receptor Targeting With ARN-509 (SPARTAN) study established that apalutamide, when added to ongoing androgen deprivation therapy (ADT), significantly delays the progression of metastatic disease or death in men with non-metastatic castrate refractory prostate cancer (nmCRPC). The common adverse events included fatigue, hypertension, rash, diarrhea, and fractures. Overall, the safety profile of apalutamide was manageable, with most adverse events being of mild to moderate severity [3].

The Targeted Investigational Treatment Analysis of Novel Anti-androgen (TITAN) trial showed that apalutamide significantly improved overall and radiographic progression-free survival in hormone-sensitive prostate cancer. The safety profile was similar to the SPARTAN trial, and notably, neither trial reported any significant myelosuppression. There was one case of febrile neutropenia (≥ grade 3) in the TITAN trial [4].

Here is a rare case of a patient with metastatic hormone-sensitive prostate cancer (mHSPC). He had no significant past medical history and presented with neutropenia and myelosuppression induced by apalutamide.

## Case presentation

A 65-year-old man with no medical history underwent radical prostatectomy in June 2021 for a pT3Nx Gleason 4+3=7 adenocarcinoma with tertiary pattern 5 and positive margins (extra-prostatic and apex). Preoperative symptoms included erectile dysfunction and perineal pain, with a prostate-specific antigen (PSA) level of 1.58 µg/L (<4.5 ug/L).

He developed biochemical progression with rising PSA in 2022 up to 4.5 µg/L. Prostate-specific membrane antigen (PSMA) PET scan showed local residual disease, and he later underwent salvage prostate bed radiotherapy (66 Gy in 33 fractions completed 24.03.22). He was also commenced on androgen deprivation therapy (ADT) with goserelin (trade name Zoladex) but experienced knee pain, pedal edema, and nipple tenderness, and ADT had to be stopped. On surveillance, PSA increased again to 2.23 µg/L; repeat PSMA PET showed a small area of nodal recurrence with no other distant disease.

He then commenced bicalutamide 50 mg once daily, and PSA fell to 0.41 µg/L. His oncologist recommended that he start ADT. As he had problems with goserelin in the past, he was started on Prostap 11.25 mg once every 12 weeks, along with a second-generation ARTA apalutamide. He was started on apalutamide 240 mg once a day, at which stage he stayed well with a performance status of zero. Full blood count at this time was normal with the following results: Hemoglobin (Hb) 141 g/L (130-180 g/L), white cell count (WCC) 4.3 x 10^9^/L (3.6-11.0 x 10^9^/L), absolute neutrophil count (ANC) 2.6 x 10^9^/L (1.8-7.5 x 10^9^/L) and platelets 298 x 10^9^/L (140-400 x 10^9^/L). He tolerated this regime well with a drop in PSA to 0.16 ug/L, and testosterone remained adequately suppressed.

**Figure 1:**
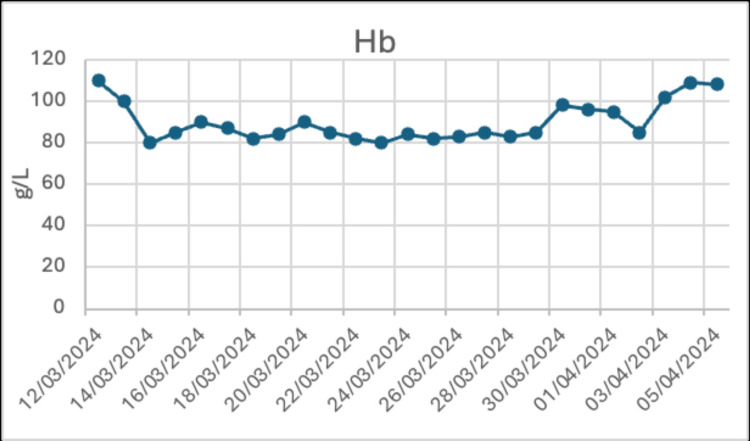
Hb trend during patient's hospital admission Hb: hemoglobin

**Figure 2:**
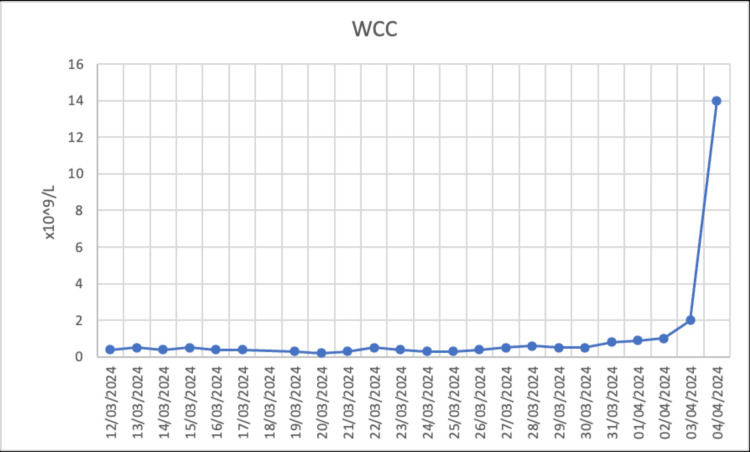
Total WCC trend during patient's hospital admission WCC: white cell count

About 62 days after starting apalutamide, he developed otalgia and elevated temperature (39.0°C) and attended the emergency department (ED). Broad-spectrum IV antibiotics (piperacillin/tazobactam) were commenced, and he was evaluated for neutropenic sepsis. Blood test at admission revealed the following: Hb 107 g/L, WCC 0.4 x 10^9^/L, ANC 0.03 x 10^9^/L, and platelets 298 x 10^9^/L. The trend of blood cell count is shown in Figures 1-2 and Tables 1-2. Apalutamide was held during admission, and he was administered granulocyte colony-stimulating factor (GCSF).

**Table 1:** Absolute neutrophil count over the course of the patient's hospital admission

Date	Absolute Neutrophil Count (ANC)
11th March 2024	0.03
15th March 2024	0.02
17th March 2024	0.01
19th March 2024	0.02
22nd March 2024	0.02
24th March 2024	0.03
26th March 2024	0.04
28th March 2024	0.02
30th March 2024	0.04
3rd April 2024	0.44
5th April 2024	12.37

**Table 2:** Platelet count over the course of the patient's hospital admission There was a dip in platelet count between 25th and 28th March.

Date	Platelet Count (×10³/μL)
11th March 2024	381
15th March 2024	298
19th March 2024	258
23rd March 2024	148
25th March 2024	121
26th March 2024	132
27th March 2024	132
28th March 2024	125
30th March 2024	257
3rd April 2024	341
5th April 2024	305

Blood cultures taken showed no source of infection. Urine culture and microscopy, chest X-ray, and viral screen all were unremarkable. MRI head and neck showed high signal and soft tissue thickening in the left external auditory canal; there was also evidence of surrounding edema. This was possibly causing pain in his left ear, leading to his admission. The biopsy of the left anterior ear canal wall lesion revealed keratotic material with inflammatory exudate and foreign body-type giant cells. No malignancy was seen on the biopsy, and the ultrasound of the neck revealed a benign reactive left submandibular lymph node with no suspicious features.

Along with neutropenia, due to the low Hb and modest thrombocytopenia, a hematology consult was requested. Bone marrow biopsy was conducted to rule out myelodysplastic syndrome. Bone marrow biopsy revealed features consistent with dysplasia. Next-generation sequencing (NGS) panel was negative for trephine findings in keeping with dysplasia.

After completion of 20 days of intravenous antibiotics, his cell count recovered. He was discharged after an inpatient stay of 30 days; the recovery of cell count rendered the diagnosis of myelodysplasia unlikely. He became afebrile after almost 15 days of hospital admission, and his cell count recovered. PSA remains well suppressed at 0.02 ug/L, and he continues ADT alone. He remains on surveillance. 

## Discussion

Neutropenia induced by chemotherapy is a significant concern because it greatly increases the risk of infections, which can lead to serious illness and even death. It also limits the doses that patients can safely receive during cancer treatment. When patients develop severe or febrile neutropenia during chemotherapy, their treatment often needs to be adjusted, with doses lowered or treatments postponed to reduce further risk [5].

As far as we know, only one case report of a patient with mHSPC who developed febrile neutropenia as a side effect of the next-generation ARAT therapy, apalutamide, has been reported. His condition fully resolved after pausing the medication and providing a brief course of GCSF [6].

A recent systematic review and network meta-analysis provided a comprehensive comparison of darolutamide, apalutamide, and enzalutamide in terms of overall survival (OS), progression-free survival, and adverse events for nmCRPC patients. Common adverse events across all three drugs included fatigue, hypertension, and musculoskeletal symptoms. Specific differences in adverse events, such as central nervous system effects with enzalutamide or rash with apalutamide, were noted. This meta-analysis suggested that the highest OS efficacy and lowest grade 3+ toxicity was for darolutamide [7].

In the phase 3 ARASENS clinical trial, 1,306 patients with metastatic HSPC were randomly assigned in a 1:1 ratio to receive darolutamide or matching placebo, both in combination with androgen-deprivation therapy and docetaxel. The occurrence of neutropenic sepsis was not significantly elevated in the darolutamide group compared to the control group receiving standard therapy alone. Additionally, severe adverse effects were 2.6% higher in the darolutamide group than in the placebo group; the most common adverse effect being neutropenia [8].

In the open-label, randomized, phase 3 ENZAMET trial, almost 1,000 men were randomly assigned to different study groups, and the participants were followed for almost more than 2.5 years to receive testosterone suppression plus either open-label enzalutamide or a standard nonsteroidal antiandrogen therapy. The occurrence of neutropenia was comparable between the two treatment groups, with a similar number of patients receiving enzalutamide and those on standard care. Nearly all of these cases developed during the initial phase of docetaxel treatment, with only two exceptions [9].

The PEACE-1 study explored the effects of combining abiraterone and prednisone with standard ADT and docetaxel for patients with newly diagnosed metastatic castration-sensitive prostate cancer. Findings showed a slightly higher rate of febrile neutropenia in patients receiving abiraterone with standard treatment and a similar rate in patients treated with both abiraterone and docetaxel compared to those receiving only standard care with docetaxel [10].

The TITAN trial demonstrated the efficacy of apalutamide in hormone-sensitive prostate cancer, showing significant improvements in overall survival and radiographic progression-free survival. A total of 525 patients were assigned to receive apalutamide plus ADT and 527 placebo plus ADT. At almost two years, a higher proportion of patients in the apalutamide group showed no radiographic disease progression compared to those in the placebo group [4].

Neutropenia is a condition wherein ANC falls below 1,500 cells/mm³. It is categorized into three levels of severity: mild (1,000-1,500 cells/mm³), moderate (500-1,000 cells/mm³), and severe (less than 500 cells/mm³). This condition can occur due to one or more underlying mechanisms, such as reduced production of neutrophils in the bone marrow, trapping or sequestering of neutrophils in certain parts of the body, or increased destruction of neutrophils in the bloodstream [11].

Notably, the trial reported only one case of febrile neutropenia, highlighting apalutamide's favorable safety profile in this patient population.

## Conclusions

This case report and literature review both emphasize the rarity and severity of apalutamide-induced neutropenia. Our case and the previously described case demonstrate the rapid onset of severe neutropenia leading to febrile neutropenia and the necessity for immediate intervention with antibiotics and GCSF, consistent with European Organization for Research and Treatment of Cancer (EORTC) guidelines. The recovery of the patient's cell counts post-discharge aligns with the literature's indication that severe neutropenia, though rare, can be managed effectively with appropriate interventions. This underscores the need for clinicians to remain vigilant about potential neutropenia when prescribing apalutamide and to consider alternative treatment options or combination therapies if severe side effects occur. To our best knowledge, this is a rare case of broader myelosuppression although anemia and thrombocytopenia are relatively less severe compared to neutropenia. The broader body of evidence supports the efficacy of apalutamide in extending metastasis-free survival in nmCRPC and overall survival and radiographic progression-free survival in hormone-sensitive prostate cancer but highlights the importance of balancing efficacy with the management of serious side effects.

## References

[1] Sung H, Ferlay J, Siegel RL, Laversanne M, Soerjomataram I, Jemal A, Bray F (2021). Global cancer statistics 2020: GLOBOCAN estimates of incidence and mortality worldwide for 36 cancers in 185 countries. CA Cancer J Clin.

[2] Patel UJ, Caulfield S (2019). Apalutamide for the treatment of nonmetastatic castration-resistant prostate cancer. J Adv Pract Oncol.

[3] Smith MR, Saad F, Chowdhury S (2018). Apalutamide treatment and metastasis-free survival in prostate cancer. N Engl J Med.

[4] Chi KN, Agarwal N, Bjartell A (2019). Apalutamide for metastatic, castration-sensitive prostate cancer. N Engl J Med.

[5] Aapro MS, Bohlius J, Cameron DA (2011). 2010 update of EORTC guidelines for the use of granulocyte-colony stimulating factor to reduce the incidence of chemotherapy-induced febrile neutropenia in adult patients with lymphoproliferative disorders and solid tumours. Eur J Cancer.

[6] Moria FA, Park CL, Saleh RR (2023). Apalutamide induced neutropenia: an unusual side effect and a literature review. J Cancer Sci Clin Ther.

[7] Wenzel M, Nocera L, Collà Ruvolo C (2022). Overall survival and adverse events after treatment with darolutamide vs. apalutamide vs. enzalutamide for high-risk non-metastatic castration-resistant prostate cancer: a systematic review and network meta-analysis. Prostate Cancer Prostatic Dis.

[8] Smith MR, Hussain M, Saad F (2022). Darolutamide and survival in metastatic, hormone-sensitive prostate cancer. N Engl J Med.

[9] Davis ID, Martin AJ, Stockler MR (2019). Enzalutamide with standard first-line therapy in metastatic prostate cancer. N Engl J Med.

[10] Fizazi K, Foulon S, Carles J (2022). Abiraterone plus prednisone added to androgen deprivation therapy and docetaxel in de novo metastatic castration-sensitive prostate cancer (PEACE-1): a multicentre, open-label, randomised, phase 3 study with a 2 × 2 factorial design. Lancet.

[11] Schwartzberg LS (2006). Neutropenia: etiology and pathogenesis. Clin Cornerstone.

